# Peripheral CD4+ naïve/memory ratio is an independent predictor of survival in non-small cell lung cancer

**DOI:** 10.18632/oncotarget.19330

**Published:** 2017-07-18

**Authors:** Peng Yang, Junhong Ma, Xin Yang, Wei Li

**Affiliations:** ^1^ Department of Thoracic Surgery, Linyi People's Hospital, Linyi 276000, China; ^2^ The Statistics Research and Consulting Laboratory, Culverhouse College of Commerce and Business Administration, The University of Alabama, Tuscaloosa, AL 35487, USA

**Keywords:** non-small cell lung cancer, T cells, CD45RA, CD45RO, survival

## Abstract

**Background:**

To investigate the clinical significance of naïve T cells, memory T cells, CD45RA+CD45RO+ T cells, and naïve/memory ratio in non-small cell lung cancer (NSCLC) patients.

**Methods:**

Pretreatment peripheral blood samples from 76 NSCLC patients and 28 age- and sex-matched healthy volunteers were collected and tested for immune cells by flow cytometry. We compared the expression of these immune cells between patients and healthy controls and evaluated their predictive roles for survival in NSCLC by cox proportional hazards model.

**Results:**

Decreased naïve CD4+ T cells, naïve CD8+ T cells, CD4+ naïve/memory ratios and CD4+CD45RA+CD45RO+ T cells, and increased memory CD4+ T cells, were observed in 76 NSCLC patients compared to healthy volunteers. Univariate analysis revealed that elevated CD4+ naïve/memory ratio correlated with prolonged progression-free survival (P=0.013). Multivariate analysis confirmed its predictive role with a hazard ratio of 0.35 (95% confidence interval, 0.19-0.75, P=0.012).

**Conclusions:**

Peripheral CD4+ naïve/memory ratio can be used as a predictive biomarker in NSCLC patients and used to optimize personalized treatment strategies.

## INTRODUCTION

Lung cancer is the most common malignant cancer and the leading cause of cancer death worldwide [1]. Surgery with or without postoperative radiochemotherapy is the standard therapy for operable non-small cell lung cancer (NSCLC) patients with stage I-III A [2, 3]. For non-operable NSCLC patients, chemotherapy, targeted therapy, radiotherapy and their combination are utilized [4, 5]. Natural innate and adaptive immunity play important roles in cancer development and interact with cancer therapeutics [6, 7]. Based on this mechanism, we hypothesized that the pretreatment systemic immune status could influence the effects of cancer therapeutics and consequently affect patients’ clinical outcomes. Many studies have investigated the prognostic value of CD4+ T cells, CD8+ T cells, regulatory T cells and B cells [8–12]. Nevertheless, very little is known of the predictive roles of peripheral naïve T cells, memory T cells and CD45RA+CD45RO+ T cells in NSCLC patients.

CD45RA+ T cells are identified as naïve T lymphocytes that have not responded to antigen; they will be activated by the stimulation of antigen and differentiate to effector cells against tumor cells. CD45RO is the marker of memory T lymphocytes which have responded to tumor antigen and will solicit immune response by the re-exposure of antigen [13–15]. T cells coexpressing CD45RA and CD45RO are special cells in the process of transformation from naïve cells to memory cells [16]. Hara et al. [17] found no differences in the proportions of naïve CD4+ T cells, memory CD4+ T cells, and the CD4+ naïve/memory ratios between NSCLC patients and healthy controls. Similarly, there were no significant differences of naïve CD8+ cells and memory CD8+ cells [18]. Two very recent studies proved that high levels of CD45RO (+) tumor-infiltrating lymphocytes were associated with better survival in lung cancer patients [19, 20].

Thus, the purpose of this study was to investigate the clinical significance of peripheral naïve T cells, memory T cells, CD45RA+CD45RO+ T cells and naïve/memory ratio in NSCLC patients.

## RESULTS

### Clinicopathologic characteristics

Clinicopathologic characteristics of 76 NSCLC patients are shown in Table 1. Among them, 41 received radical surgery and 35 were treated with radiotherapy combined with chemotherapy/targeted therapy. Tumors with epidermal growth factor receptor (EGFR) mutation occurred in 4 patients; anaplastic lymphoma kinase (ALK) gene fusion occurred in 2 patients. nineteen patients had evidence of distant metastases at diagnosis; there were 5 with lung metastases, 3 with brain metastases, 6 with bone metastases, and 5 with adrenal metastasis.

**Table 1:** Clinicopathologic characteristics of 76 NSCLC patients

Parameter	N=76(%)
Age(years)	64.5(35-87)
Gender	
Male	54(71.1)
Female	22(28.9)
ECOG	
0	32(42.1)
1-2	44(57.9)
Smoking history	
Yes	50(65.8)
No	26(34.2)
Histology	
Squamous cell carcinoma	37(48.7)
Adenocarcinoma	39(51.3)
Differentiation	
Well/moderate	48(63.2)
Poor	23(30.3)
Unknown	5(6.5)
T stage	
1	20(26.3)
2	32(42.1)
3	11(14.5)
4	13(17.1)
N stage	
0	21(27.6)
1	24(31.6)
2	19(25.0)
3	12(15.8)
Clinical stage (TNM)	
I	8(10.5)
II	16(21.1)
III	33(43.4)
IV	19(25.0)

NSCLC=non-small cell lung cancer, ECOG=Eastern Cooperative Oncology Group.

### The expression of immune cells in 76 NSCLC patients

**Figure 1:**
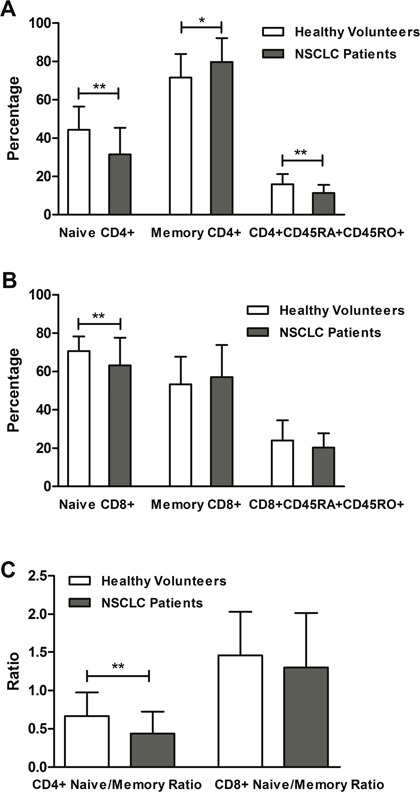
Differences in the proportions of CD45RA and CD45RO marked T cells between NSCLC patients and healthy controls In NSCLC, there are **(A)** decreased naïve CD4+ T cells and CD4+CD45RA+CD45RO+ T cells, and increased memory CD4+ T cells; **(B)** reduced naïve CD8+ T cells; **(C)** low CD4+ naïve/memory ratio. ^*^P < 0.05; ^**^ *P* < 0.01.

Lung cancer patients expressed significantly decreased proportions of naïve CD4+ T cells, naïve CD8+ T cells, CD4+ naïve/memory ratios, and CD4+CD45RA+CD45RO+ T cells and increased memory CD4+ T cells compared to healthy controls (Figure 1, Table 2). In addition, a similar trend occurred in CD8+ naïve/memory ratios, CD8+CD45RA+CD45RO+ T cells and memory CD8+ T cells, but the differences did not reach statistical significance.

**Table 2:** Differences of immune cells between NSCLC patients and healthy volunteers

Variable	Healthy volunteers	NSCLC	*P-value*
	Mean±SD	Mean±SD	
Naïve CD4+	44.26±12.21	31.46±15.26	0.002
Memory CD4+	71.52±12.35	79.83±13.71	0.025
CD4+CD45RA+CD45RO+	15.85±5.32	11.38±4.49	0.001
CD4+ naïve/memory ratio	0.67±0.31	0.44±0.34	0.009
Naïve CD8+	70.66±7.62	61.15±15.02	0.045
Memory CD8+	53.35±14.35	57.03±16.3	0.451
CD8+CD45RA+CD45RO+	24.06±10.46	20.28±7.79	0.117
CD8+ naïve/memory ratio	1.46±0.57	1.30±0.73	0.450

NSCLC=non-small cell lung cancer, SD=standard deviation.

### Correlation of immune cells with clinicopathologic characteristics

Table 3 summarizes the relationships between immune cells and clinicopathological characteristics in the 76 NSCLC patients. In never-smokers, high proportions of naïve CD4+ T cells and low memory CD4+ T cells were observed. Similarly, there was a trend in younger patients for these findings although they did not achieve statistical significance. In patients with good performance status, decreased naïve CD8+ T cells and CD8+ naïve/memory and increased memory CD8+ T cells were found.

**Table 3:** Associations between immune parameters and clinicopathologic parameters in 76 NSCLC patients

Variable	Naïve CD4+		Memory CD4+		CD4+CD45RA+CD45RO+		CD4+ naïve/memory ratio		Naïve CD8+		Memory CD8+		CD8+CD45RA+CD45RO+		CD8+ naïve/memory ratio	
	Mean±SD	P	Mean±SD	P	Mean±SD	P	Mean±SD	P	Mean±SD	P	Mean±SD	P	Mean±SD	P	Mean±SD	P
Age(years)																
<64.5	39.15±15.40	0.051	73.47±14.97	0.062	12.69±5.29	0.377	0.61±0.41	0.056	65.32±19.40	0.216	54.43±19.64	0.173	19.82±6.18	0.646	1.52±1.00	0.054
≥64.5	28.90±14.19		82.32±12.13		11.29±4.01		0.39±0.26		58.80±11.73		62.21±13.77		21.08±8.67		1.03±0.45	
Gender																
Female	35.98±12.98	0.417	76.77±11.68	0.531	12.84±4.26	0.396	0.50±0.26	0.685	58.98±18.76	0.597	59.49±20.45	0.984	18.53±5.79	0.324	1.22±0.78	0.97
Male	31.30±16.09		80.03±14.52		11.4±4.59		0.45±0.37		61.99±13.66		59.36±14.88		21.43±8.40		1.21±0.72	
ECOG																
0	30.52±11.86	0.547	80.94±10.46	0.557	11.53±4.65	0.794	0.40±0.22	0.395	53.54±14.29	0.020	67.86±13.50	0.017	21.48±6.81	0.626	0.85±0.34	0.007
1-2	33.77±17.02		78.09±15.37		11.95±4.50		0.50±0.39		65.46±13.92		54.62±16.03		20.14±8.41		1.41±0.81	
Smoking history																
No	41.13±13.95	0.024	72.51±12.87	0.053	13.69±5.08	0.093	0.61±0.29	0.084	66.68±19.77	0.146	54.82±22.44	0.269	21.55±8.55	0.646	1.58±1.04	0.042
Yes	28.84±14.51		82.03±13.27		10.96±4.03		0.40±0.34		58.72±12.08		61.41±12.80		20.22±7.59		1.05±0.48	
Histology																
SCC	27.51±14.20	0.102	82.7±11.53	0.246	10.29±4.55	0.054	0.36±0.24	0.138	58.25±14.52	0.447	62.86±14.35	0.394	21.18±8.57	0.772	1.03±0.51	0.282
ADC	35.82±14.59		77.3±15.05		13.20±3.84		0.53±0.40		62.15±14.97		58.14±17.47		20.37±7.54		1.29±0.85	
Differentiation																
Well/Moderate	33.56±15.52	0.243	78.39±13.83	0.281	12.02±5.15	0.544	0.49±0.37	0.325	60.44±15.45	0.643	60.95±15.50	0.614	21.48±7.97	0.850	1.12±0.56	0.332
Poor	26.03±14.61		84.69±13.99		10.82±3.12		0.35±0.28		63.38±14.41		57.43±19.92		20.83±8.98		1.41±1.08	
T stage																
1	30.23±14.90	0.626	82.92±11.79	0.381	13.25±5.70	0.307	0.40±0.29	0.517	63.24±14.19	0.662	54.33±17.17	0.326	17.71±8.14	0.235	1.35±0.74	0.536
2-4	33.28±15.56		78.03±14.21		11.38±4.11		0.49±0.35		60.56±15.45		60.85±16.07		21.46±7.64		1.17±0.73	
N stage																
0	31.93±16.66	0.882	80.65±16.09	0.705	12.71±5.64	0.491	0.48±0.48	0.893	61.06±11.55	0.982	57.93±15.06	0.760	19.17±7.34	0.524	1.17±0.52	0.850
1-3	32.82±15.10		78.61±13.12		11.49±4.12		0.46±0.29		61.19±16.21		59.89±16.94		21.11±8.01		1.22±0.79	
Clinical stage (TNM)																
I-III	31.59±13.75	0.556	79.85±11.23	0.636	11.52±5.02	0.582	0.43±0.25	0.310	61.42±15.38	0.875	59.49±15.90	0.960	20.99±8.45	0.676	1.18±0.63	0.757
IV	34.90±18.78		77.46±18.71		12.43±3.06		0.55±0.48		60.55±14.87		59.19±17.98		19.79±6.34		1.27±0.94	

NSCLC=non-small cell lung cancer, SD=standard deviation, ECOG=Eastern Cooperative Oncology Group, SCC=squamous cell carcinoma, ADC=adenocarcinoma.

### Increased CD4+ naïve/memory ratio associated with better progression-free survival

**Figure 2:**
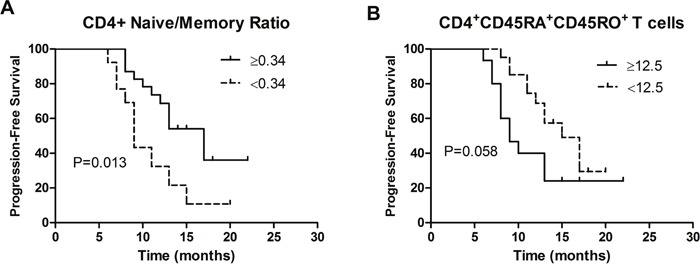
Progression-free survival of 76 NSCLC patients stratified by **(A)** CD4+ naïve/memory ratio; **(B)** CD4+CD45RA+CD45RO+ T cells.

Univariate analysis of immune parameters and clinicopathologic parameters are shown in Table 4. Interestingly, patients with a high level of CD4+ naïve/memory ratio had a better PFS (HR = 0.47, 95%CI = 0.20-0.92, *P* = 0.013, Figure 2A). In contrast, elevated CD4+CD45RA+CD45RO+ T cells predicted poor PFS with a robust trend toward significance (HR = 2.71, 95%CI = 1.15-6.36, *P* = 0.058, Figure 2B). Besides, patients with an increase of memory CD4+ T cells may have a poor PFS (HR = 1.82, 95%CI = 0.78-4.25, *P* = 0.138, Table 4).

**Table 4:** Progression free survival of 76 NSCLC patients stratified by immune parameters and clinicopathologic parameters

Variable	Median survival (months)	HR (95%CI)	*P-value*
Naïve CD4+			
<median	10	1	0.583
≥median	13	0.80(0.35-1.85)	
Memory CD4+			
<median	15	1	0.138
≥median	9	1.82(0.78-4.25)	
CD4+CD45RA+CD45RO+			
<12.5	15	1	0.058
≥12.5	9	2.71(1.15-6.36)	
CD4+ naïve/memory ratio			
<0.34	9	1	0.013
≥0.34	17	0.47(0.20-0.92)	
Naïve CD8+			
<median	13	1	0.808
≥median	13	1.11(0.47-2.59)	
Memory CD8+			
<median	13	1	0.726
≥median	13	1.15(0.50-2.67)	
CD8+CD45RA+CD45RO+			
<median	12	1	0.806
≥median	15	0.90(0.39-2.11)	
CD8+ naïve/memory ratio			
<median	13	1	0.567
≥median	13	0.79(0.34-1.84)	
Gender			
Female	15	1	0.156
Male	12	1.85(0.68-6.35)	
Age(years)			
<64.5	13	1	0.682
≥64.5	15	0.86(0.29-1.58)	
Smoking status			
No	15	1	0.583
Yes	13	1.25(0.68-3.15)	
Differentiation			
Well and moderate	15	1	0.006
Poor	9	3.95(2.35-12.37)	
ECOG			
0	17	1	0.018
1-2	12	3.46(1.28-8.24)	
T stage			
1	15	1	0.482
2-4	13	1.82(0.79-6.25)	
N stage			
0	17	1	0.026
1-3	13	3.28(1.13-9.67)	
Clinical stage (TNM)			
I-III	15	1	0.004
IV	9	3.68(1.45-8.47)	

NSCLC=non-small cell lung cancer, HR=hazard ratio, CI=confidence interval, ECOG=Eastern Cooperative Oncology Group performance status.

In addition to immune parameters, we found other parameters associated with clinical outcomes. Tumor differentiation, nodal stage, clinical stage and the Eastern Cooperative Oncology Group performance status correlated with the patients’ clinical outcome (Table 4).

### CD4+ naïve/memory ratio independently correlated with progression-free survival

Multivariate cox proportional hazard models revealed that increased CD4+ naïve/memory ratio was independently associated with better PFS (HR = 0.35, 95%CI = 0.19-0.75, *P* = 0.012, Table 5). In addition, nodal stage and clinical stage were independently correlated with the progression-free survival of 76 NSCLC patients.

**Table 5:** Multivariate cox proportional hazard models for progression free survival of 76 NSCLC patients

Variable	HR	95%CI	*P-value*
N stage			
1-3 vs 0	10.79	2.43-47.85	0.002
Clinical stage			
IV vs I-III	4.61	1.34-15.91	0.016
ECOG			
1-2 vs 0	2.77	0.82-9.35	0.135
Differentiation			
Poor vs Well and moderate	3.34	0.84-13.33	0.087
CD4+ naïve/memory ratio			
≥0.34 vs <0.34	0.35	0.19-0.75	0.012

NSCLC=non-small cell lung cancer, ECOG=Eastern Cooperative Oncology Group.

## DISCUSSION

To the best of our knowledge, this is a study with the largest sample size to investigate the predictive roles of peripheral naïve/memory ratio, naïve cells, memory cells, and CD45RA+CD45RO+ T cells in patients with NSCLC. The most important findings were that elevated CD4+ naïve/memory ratio was independently correlated with prolonged PFS. Moreover, lower percentages of naïve CD4+ T cells, naïve CD8+ T cells, CD4+ naïve/memory ratios, and CD4+CD45RA+CD45RO+ T cells, and increased memory CD4+ T cells, occurred in NSCLC patients compared to healthy controls.

Hara et al. [17] investigated the correlation between CD4+ naïve/memory ratio and survival after surgery in NSCLC patients and proved this possible relationship; however, there were only 8 patients in each group. In the present study, we found that elevated CD4+ naïve/memory ratio was independently associated with prolonged PFS in NSCLC patients; elevated peripheral memory CD4+ cells may indicate poor survival, which is contrary to the role of CD45RO+ tumor-infiltrating lymphocytes in tumor tissues [19–21]. Several possibilities may explain above phenomena. First, in tumor tissues, memory cells elicited robust immune response to attack tumor cells [13]. However, memory CD4+ cells in peripheral blood showed lower proliferation than primary responding cells when they re-encountered antigen and failed to elicit robust immune response [22–24]. In contrast, naïve T cells were more capable of proliferation and anti-tumor efficiency [25]. Besides, high levels of CD45RO+ tumor-infiltrating lymphocytes were associated with low tumor stage and invasion [26]. Correlations between elevated CD4+CD45RA+CD45RO+ cells and poor survival were observed with a trend in univariate analysis. CD4+CD45RA+CD45RO+ cells transitioned from naïve to memory cells after stimulation by tumor antigen; increased proportions of these cells may suggest higher tumor malignancy and invasion.

Furthermore, we found no predictive roles of naïve CD8+ T cells, memory CD8+ T cells, CD8+ naïve/memory ratio, and CD8+CD45RA+CD45RO+ T cells, which agrees with previous studies [17, 18]. Decreased percentages of naïve CD4+ T cells, naïve CD8+ T cells, CD4+ naïve/memory ratios, and CD4+CD45RA+CD45RO+ T cells and increased memory CD4+ T cells were observed in NSCLC patients, suggesting that the immune system was activated against cancer. A previous study has not found these results with statistical significance, which may have been accounted for by the small number of patients and early tumor grade [17]. In nonsmokers and young patients, increased percentages of naïve CD4+ T cells and decreased memory CD4+ T cells were observed, which were consistent with tumor characteristics of these patients.

The limitations of this study include small sample size, different clinical stages, different treatment strategies and a short follow-up time that results in the lack of detailed information on overall survival. Despite these weaknesses, we documented that CD4+ naïve/memory ratio independently predicted tumor progression. Moreover, decreased proportions of naïve CD4+ T cells, naïve CD8+ T cells, CD4+ naïve/memory ratio and CD4+CD45RA+CD45RO+ T cells, and increased memory CD4+ T cells were observed in NSCLC patients compared to healthy controls.

In summary, peripheral CD4+ naïve/memory ratio may provide an independent predictor of tumor progression in NSCLC patients. This information may provide opportunities to select NSCLC patients who would have poor survival, and optimize treatment strategies for them.

## MATERIALS AND METHODS

### Patients selection

We prospectively included 76 NSCLC patients with histological confirmation, who were treated in Linyi People's Hospital. The inclusion criteria for patients were as follows: They had not received anti-tumor therapies or other therapies that influence patients’ immune status before enrollment and were over 18 years old; they had no infections, other cancers, immune system related diseases or transplant history. Twenty-eight age- and sex-matched healthy volunteers were included. Clinicopathologic characteristics of the enrolled NSCLC patients were collected from the electronic medical record system in our hospital. Clinical stage was evaluated by American Joint Committee on Cancer-7 criteria. Informed consent was obtained from all patients and volunteers. The present study was approved by the Ethical Committee of Linyi People's Hospital. All methods in the present study were approved by the committee and performed in accordance with relevant guidelines and regulations.

### Treatment protocol and follow-up

Early stage patients received radical surgery and lymph node dissection; advanced patients received radiotherapy combined with chemotherapy/targeted therapy concurrently or consecutively. First-line chemotherapy was with platinum-based agents; gefitinib, erlotinib, and crizotinib were used for targeted therapy. Radiotherapy was delivered with 60-66 Gy/30-33 fractions by linear accelerator. Patients were followed up regularly per 3 months after treatment. Follow-up data collection ended on April 16, 2017 or the time of death of the patient. The median follow-up was 13 months (range 6-22.5 months). The primary endpoint was PFS, which was defined as the interval between the date of initial therapy and the date of the first RECIST- (Response Evaluation Criteria in Solid Tumors) 1.1 [27] defined progression, death, or loss to follow-up.

### Detection of immune cells

Fresh peripheral blood samples obtained from patients prior to cancer treatment and from volunteers were stored in EDTA anticoagulation tubes before testing. Four specific monoclonal antibodies (mAbs) against CD4 (FITC and APC), CD8 (FITC and APC), CD45RA (FITC), CD45RO (PE) were used to differentiate immune cells. Initially, 200μl blood was mixed with the above mAbs and incubated in the dark at room temperature. By using FACS lysing solution (BD Biosciences, San Jose, CA, USA), red blood cells in the mix were lysed and then washed twice with phosphate buffered saline (PBS). The residual white blood cells were analyzed by flow cytometry and the percentages of these immune cells were calculated by FlowJo Version 10.0 data analysis software (FlowJo, Ashland, OR, USA).

Immune cells were identified as follow: naïve CD4+ T cells (CD4+CD45RA+), memory CD4+ T cells (CD4+CD45RO+), naïve CD8+ T cells (CD8+CD45RA+), memory CD8+ T cells (CD8+CD45RO+), CD4+CD4 5RA+CD45RO+ T cells (CD4+CD45RA+CD45RO+) and CD8+CD45RA+CD45RO+ T cells (CD8+CD45RA+ CD45RO+). The percentages of immune cells were expressed as proportions of CD4+/CD8+ T cells.

### Statistical analysis

Percentages of immune cells were expressed with mean±standard deviation (SD). Differences of immune cells between NSCLC patients and controls, and relationships between patient characteristics and immune cells were determined by the Student's *t*-test. The Kaplan-Meier analysis was employed to estimate PFS and compare PFS between groups by log-rank test. The Cox proportional hazards model was performed to determine hazard ratios (HRs) and 95% confidence intervals (CIs). Variables of *P* < 0.05 in univariate analysis were enrolled in multiple analysis. Data analyses were performed using the Statistical Package for Social Sciences, Version 22.0 (IBM Corporation, Armonk, NY, USA) and GraphPad Version 5.0 (GraphPad Software Inc., San Diego, CA, USA); *P* < 0.05 was defined as statistically significant.
